# A genome-wide dsRNA library screen for *Drosophila* genes that regulate the GBP/phospholipase C signaling axis that links inflammation to aging

**DOI:** 10.1186/s13104-018-3996-z

**Published:** 2018-12-13

**Authors:** Eui Jae Sung, Stephen B. Shears

**Affiliations:** Inositol Signaling Group, Signal Transduction Laboratory, National Institute of Environmental Health Sciences, National Institutes of Health, 111 T.W. Alexander Drive, Research Triangle Park, NC 27709 USA

**Keywords:** Cytokine, Inflammation, Metabolism, Calcium-signaling, G-proteins, Receptor

## Abstract

**Objective:**

Invertebrates are productive models for understanding how inflammation, metabolism and aging are intertwined. We have deployed a dsRNA library screen to search for genes in *Drosophila melanogaster*—and hence identify human orthologs—that encode participants in a G-protein coupled, Ca^2+^-signaling pathway that regulates inflammation, metabolism and lifespan.

**Results:**

We analyzed receptor-dependent, phospholipase C/Ca^2+^ signaling responses to the growth-blocking peptide (GBP) cytokine in *Drosophila* S3 cells plated in 384-well plates containing dsRNAs that target approximately 14,000 *Drosophila* genes. We used Z-scores of < − 3 or > + 3 to define gene hits. Filtering of ‘housekeeping’ genes from these hits yielded a total of 82 and 61 *Drosophila* genes that either down-regulate or up-regulate Ca^2+^-signaling, respectively; representatives from these two groups were validated. Human orthologs of our hits may be modulators of Ca^2+^ signaling in general, as well as being candidates for acting in molecular pathways that interconnect aging and inflammation.

## Introduction

A systems-level understanding of cytokine-mediated, inter-tissue signaling can help to generate fundamental insight into links between longevity, metabolism and inflammation [1]. Research with human subjects indicates that aging is affected by the balance between circulating pro- and anti-inflammatory factors [2] which are subject to various environmental influences, among which calorific restriction receives particular attention [3]. However, little is known concerning the precise nature of the molecular entities and pathways that intertwine these biological phenomena in humans; most work in this area uses animal models.

Invertebrates are productive, genetically-tractable models for understanding how inflammation and aging are inter-related in humans [1, 4]. Recent work has established that a *Drosophila* cytokine, growth-blocking peptide (GBP), interconnects longevity, inflammation and dietary influences, through activation of the phospholipase C (PLC)/Ca^2+^ signaling cascade [5, 6]. The spatiotemporal control of stimulus-activated Ca^2+^ dynamics by PLC is a multiplex cellular process that coordinates three fundamental activities: maintenance of basal Ca^2+^ pools, release of Ca^2+^ from intracellular stores, and Ca^2+^ fluxes across the plasma membrane [7]. A complete understanding of the integration of these processes requires characterization of the networking of a multitude of individual regulatory components; this degree of systems-biology insight has not yet been attained. Some important information has been obtained by exploiting the amenability of *Drosophila* to the application of dsRNA libraries [8, 9]. However, the latter studies were technically limited to identification of the subset of proteins that impact Ca^2+^ fluxes across the plasma membrane; in the current study, we have sought to widen the knowledge-base through identification of regulatory factors that may impact release of Ca^2+^ release from intracellular stores *and* Ca^2+^ fluxes into the cell, i.e., total Ca^2+^ mobilization ([Ca^2+^]_T_).

For this work we deployed a fluorophore-based assay for GBP-mediated Ca^2+^ mobilization in *Drosophila* S3 cells in a 384-well, high-throughput screening format [5]. In that latter study, we screened a relative small dsRNA sub-library that targets 1729 genes known (or computationally predicted) to encode transmembrane proteins. In that way, we identified several integral membrane proteins that contribute to GBP-mediated Ca^2+^ release [5]. Nevertheless, that particular dsRNA sub-library only covered 12% of all *Drosophila* genes. In the current study, we performed a genome-wide dsRNA screen (almost 14,000 genes) so as to identify a more complete set of regulatory factors. This approach could be particularly beneficial in the context of diseases where changes in Ca^2+^-signaling are causative, since that can provide identify novel therapeutic targets [7].

## Main text

### Methods

#### Screening of the dsRNA library

We purchased a genome-wide dsRNA library (v2.0) from the DRSC/TRiP Functional Genomics Resources (https://fgr.hms.harvard.edu/). This library contains 66 × 384 well-plates that target almost 14,000 *Drosophila* genes with either one or two dsRNA constructs. The library is provided in duplicate (i.e., as a total of 132 plates). The biological readout during the screening was the total, GBP-induced change in fluorescence (equivalent to [Ca^2+^]_T_) emitted from a genetically encoded Ca^2+^ sensor in *Drosophila* S3 cells, in response to the addition of 500 nM GBP (added after 5-days pre-treatment with the dsRNAs [5]. A FLIPR^TETRA^ (Molecular Devices) was used to simultaneously record [Ca^2+^]_T_ in every well of a 384-well plate. We describe these procedures in more detail in our previous study with a small dsRNA sub-library that targets 1729 genes [5].

#### Evaluation and deposition of the screening data

Z-scores were calculated for every unique dsRNA. A ‘positive’ gene-hit for any single dsRNA was defined by a reduction in [Ca^2+^]_T_ that yielded a Z-score of less than − 3; the Z-score of a dsRNA = ([[Ca^2+^]_T_ of each dsRNA] − [average [Ca^2+^]_T_ of plate])/[SD of plate [Ca^2+^]_T_]. This strict disambiguation approach is designed to avoid false positives. Additionally, we describe some unusual, ‘negative’ gene-hits: dsRNAs that yielded an increase in [Ca^2+^]_T_ (with a Z-score greater than 3).

**Fig. 1:**
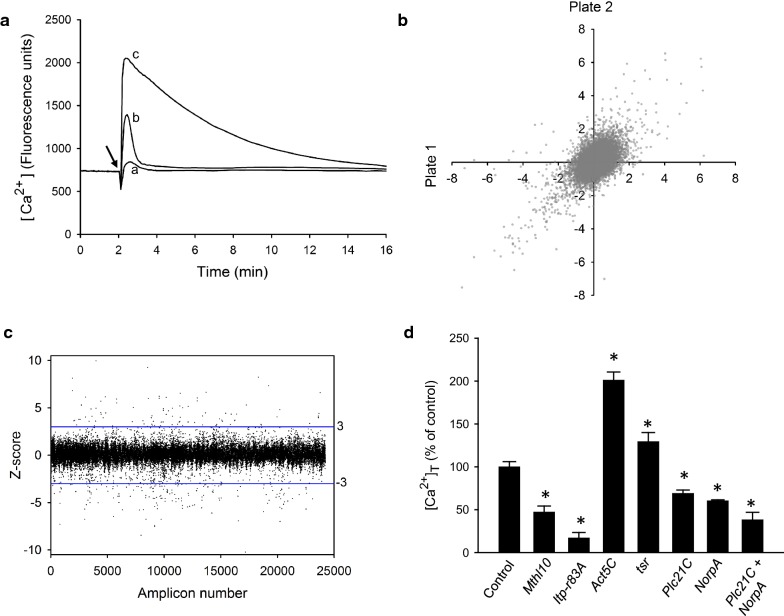
Identification and validation of genes that regulate GBP/PLC-mediated Ca^2+^ signaling. **a** Representative Ca^2+^-signaling responses from *Drosophila* S3 cells in plate 116B88 that were pre-treated with either control dsRNAi (traces ‘a’ and ‘c’) or our dsRNAi construct against *Itp*-*r83A* (trace ‘b’) before addition of either vehicle (trace ‘a’) or 500 nM GBP (traces ‘b’ and ‘c’). **b** reproducibility of dsRNA replicates obtained from 23 plates assayed in duplicate. **c** Z-scores from all amplicons. **d** Effects upon [Ca^2+^]_T_ after treatment of cells with our dsRNA constructs against the indicated genes; n = 4; *p < 0.001

We encountered more plate-to-plate variability than in our previous study with the transmembrane dsRNA sub-library [5]. In the current study, for a number of plates, the cells in individual wells exhibited values for [Ca^2+^]_T_ that clustered atypically close to that of the control, raising the possibility that knock-down efficiencies were abnormally low. We did not establish the cause of this variability, although it was not specific to any particular plate barcodes. We resolved this problem in the following manner: the *Itp*-*r83A* gene was one of our initial, and statistically most significant hits (Table 1); we independently validated this hit using alternate dsRNA constructs (Fig. 1a). Thus, we decided to use this as an internal control. This led us to establish that an adequate range of [Ca^2+^]_T_ signals within a plate was typically observed whenever our *Itp*-*r83A* dsRNA caused at least 60% inhibition of GBP-mediated Ca^2+^ signaling. Thus, all data were discarded from any plate in which the inhibition by the internal *Itp*-*r83A* dsRNA control did not reach the 60% cut-off. We were able to procure new plates from the vendor to replace most of those plates that we discarded, and eventually we performed enough assays to screen every dsRNA in the genome-wide library at least once. Previous studies that used similar plates to screen for gene knock-downs that target Ca^2+^ entry into the cell [8, 9] do not state if a similar problem was experienced. However, a different version of the dsRNA library (i.e., v1) was deployed in those earlier studies; we used v2.

**Table 1:** Filtered list of gene knock-downs that reduced GBP-mediated Ca^2+^ mobilization

Flybase ID	Gene	Amplicon #	Mean Z-score
FBgn0033246	ACC	DRSC06059	− 3.15
FBgn0025725	alphaCOP	DRSC08706	− 5.27
FBgn0003884	alphaTub84B	DRSC12622; DRSC25011	− 3.32; − 3.09
FBgn0013749	Arf102F	DRSC17195	− 4.18
FBgn0033062	Ars2	DRSC04893	− 3.06
FBgn0010217	ATPsynbeta	DRSC17194	− 3.48
FBgn0014127	barr	DRSC03488	− 3.52
FBgn0025724	beta’COP	DRSC03492; DRSC26650	− 4.27; − 5.03
FBgn0259876	Cap-G	DRSC21805	− 4.04
FBgn0022213	Cas	DRSC26857	− 3.13
FBgn0022942	Cbp80	DRSC18450	− 3.12
FBgn0012058	Cdc27	DRSC11112	− 3.3
FBgn0030510	CG12177	DRSC19437	− 7.01
FBgn0033429	CG12929	DRSC06242	− 6.12
FBgn0031023	CG14200	DRSC19555	− 3.02
FBgn0266917	CG16941	DRSC15166	− 3.92
FBgn0031498	CG17260	DRSC00497	− 8.46
FBgn0083978	CG17672	DRSC09230	− 3.49
FBgn0035205	CG2469	DRSC08562	− 3.14
FBgn0031266	CG2807	DRSC00535	− 5.48
FBgn0031493	CG3605	DRSC00619	− 5.79
FBgn0058198	CG40198	DRSC21068	− 3.12
FBgn0035983	CG4080	DRSC10398	− 6.77
FBgn0086758	chinmo	DRSC28547	− 3.26
FBgn0259993	CR42491	DRSC25025	− 4.52
FBgn0028836	CSN7	DRSC06807; DRSC06808	− 3.03; − 3.07
FBgn0025455	CycT	DRSC11124	− 3.6
FBgn0086687	Desat1	DRSC23578	− 5.23
FBgn0260635	Diap1	DRSC11404	− 4.59
FBgn0039183	Dis3	DRSC16034	− 3.99
FBgn0004638	drk	DRSC07606	− 3.52; − 3.54
FBgn0034975	enok	DRSC04096	− 3.07
FBgn0033859	fand	DRSC39027	− 4.62
FBgn0004656	fs(1)h	DRSC29017	− 3.39
FBgn0004435	Galphaq	DRSC07432	− 3.36
FBgn0001105	Gbeta13F	DRSC20247	− 5.32
FBgn0014189	Hel25E	DRSC03342	− 3.76
FBgn0053818	His3:CG33818	DRSC21267	− 4.05
FBgn0015393	hoip	DRSC03546	− 5.3
FBgn0001218	Hsc70-3	DRSC25105	− 3.05
FBgn0266599	Hsc70-4	DRSC29729	− 3.32
FBgn0010051	Itp-r83A	DRSC12354	− 6.95
FBgn0004378	Klp61F	DRSC28179	− 3.31
FBgn0001491	l(1)10Bb	DRSC20346	− 3.32
FBgn0001986	l(2)35Df	DRSC03560	− 4.51
FBgn0011640	lark	DRSC11362; DRSC25108	− 4.28; − 5.71
FBgn0035889	mkg-p	DRSC10777	− 3.13
FBgn0032921	Mpp6	DRSC03169	− 3.77
FBgn0035132	mthl10	DRSC39158	− 3.58
FBgn0086707	ncm	DRSC02179	− 5.76
FBgn0026401	Nipped-B	DRSC07815; DRSC29151	− 3.27; − 3.01
FBgn0014366	noi	DRSC12383	− 3.03
FBgn0005648	Pabp2	DRSC07501	− 3.32
FBgn0259214	PMCA	DRSC17154	− 4.59
FBgn0010590	Prosbeta1	DRSC07159	− 7.45
FBgn0026380	Prosbeta3	DRSC16801	− 3.72
FBgn0032006	Pvr	DRSC36840	− 5.7
FBgn0003189	r	DRSC19813; DRSC19814	− 4.38; − 4.44
FBgn0020255	Ran	DRSC28160	− 4.51
FBgn0003205	Ras85D	DRSC39132	− 3.42
FBgn0031868	Rat1	DRSC02044	− 3.31
FBgn0011704	RnrS	DRSC07533; DRSC23541	− 3.50; − 3.79
FBgn0010173	RpA-70	DRSC16830	− 3.63
FBgn0015805	Rpd3	DRSC08696	− 3.32
FBgn0262955	RpII140	DRSC16831	− 4.09
FBgn0003277	RpII215	DRSC20280	− 3.11
FBgn0028694	Rpn11	DRSC03422	− 5.4
FBgn0028689	Rpn6	DRSC07541	− 4.56
FBgn0028688	Rpn7	DRSC16841	− 4.81
FBgn0002787	Rpn8	DRSC04624	− 3.37
FBgn0028684	Rpt5	DRSC16842	− 3.02
FBgn0038269	Rrp6	DRSC16223	− 3.87
FBgn0262601	SmB	DRSC03437	− 3.07
FBgn0261789	SmD2	DRSC12536	− 3.88
FBgn0261790	SmE	DRSC02680	− 4.03
FBgn0028982	Spt6	DRSC18836	− 4.58
FBgn0038810	Srp72	DRSC15800	− 3.69
FBgn0045073	Stim	DRSC20158	− 4.54
FBgn0003575	su(sable)	DRSC18839	− 3.15
FBgn0030365	Tango4	DRSC23475	− 3.85
FBgn0035713	velo	DRSC08841	− 3.45
FBgn0003978	vls	DRSC02101	− 6.77

The genes listed are the ‘hits’ that *reduced* GBP-mediated Ca^2+^ mobilization (i.e., Z-score > 3), after filtering of housekeeping genes (see “Methods”)

The complete list of Z-scores is available at http://www.flyrnai.org/. Here, we tabulate two filtered hit-lists (Tables 1 and 2), from which we have removed all hits that could reasonably be predicted to non-specifically affect protein synthesis, by virtue of their having the following categorizations in the Gene Ontology Database (http://www.geneontology.org/): mRNA splicing (GO:0000398); structural constituent of ribosome (GO:0003735); translation initiation factor activity (GO:0003743); transcription factor activity, sequence-specific DNA binding (GO:0003700); RNA polymerase II transcription cofactor activity (GO:0001104); RNA polymerase II activity (GO:0001055); DNA-directed RNA polymerase activity (GO:0003899); transcription factor binding (GO:0008134); transcriptional activator activity, RNA polymerase II core promoter proximal region sequence-specific binding (GO:0001077); translation (GO:0006412); translation elongation factor activity (GO:0003746).

**Table 2:** Filtered list of gene knock-downs that increased GBP-mediated Ca^2+^ mobilization

Flybase ID	Gene name	Amplicon #	Mean Z-score
FBgn0027084	Aats-lys	DRSC25618	3.62
FBgn0000043	Act42A	DRSC04835	4.46
FBgn0000044	Act57B	DRSC04042	5.98
FBgn0000042	Act5C	DRSC17723; DRSC25024	7.32; 8.13
FBgn0000045	Act79B	DRSC11604	3.69
FBgn0087035	AGO2	DRSC10847; DRSC40785	4.89; 3.95
FBgn0041188	Atx2	DRSC40788	4.40
FBgn0000212	brm	DRSC11330; DRSC26226	4.33; 9.95
FBgn0029856	CG11700	DRSC23384	3.67
FBgn0033507	CG12909	DRSC27706	3.07
FBgn0039601	CG1523	DRSC15033; DRSC26315	3.49; 3.10
FBgn0035569	CG15876	DRSC08484	3.71
FBgn0067622	LSm-4	DRSC02845	3.44
FBgn0032240	CG17768	DRSC02845	3.44
FBgn0034325	CG18539	DRSC06766	3.12
FBgn0034326	CG18540	DRSC06767	3.10
FBgn0011824	CG4038	DRSC25331	3.79
FBgn0036991	CG5872	DRSC11785	3.64
FBgn0035872	CG7185	DRSC10781	3.49
FBgn0259236	comm3	DRSC09995	3.24
FBgn0031831	COX5BL	DRSC27300	6.17
FBgn0263093	CR43361	DRSC23926	3.19
FBgn0033260	Cul4	DRSC27185	4.46
FBgn0086901	cv-c	DRSC27016	4.33
FBgn0002413	dco	DRSC16929	3.26
FBgn0034246	Dcr-2	DRSC29436	3.53
FBgn0260049	flr	DRSC09787	3.11
FBgn0034964	IntS1	DRSC04343; DRSC27942	9.26; 5.92
FBgn0026679	IntS4	DRSC17810	5.00
FBgn0036570	IntS9	DRSC10493	3.52
FBgn0004419	me31B	DRSC03569	3.30
FBgn0035473	mge	DRSC08721	3.58
FBgn0027378	MRG15	DRSC16731	4.68
FBgn0085417	natalisin	DRSC23390	3.78
FBgn0032725	Nedd8	DRSC02092	6.04
FBgn0041102	ocn	DRSC17020	3.25
FBgn0020626	Osbp	DRSC16779	3.21
FBgn0044826	Pak3	DRSC26832	4.48
FBgn0050382	CG30382	DRSC07515	3.16
FBgn0263121	Prosalpha1	DRSC07515	3.16
FBgn0086134	Prosalpha2	DRSC28078	5.52
FBgn0261394	Prosalpha3	DRSC04644	3.19
FBgn0004066	Prosalpha4	DRSC20271	4.03
FBgn0020618	Rack1	DRSC03405; DRSC23796	5.15; 6.14
FBgn0033897	Rcd1	DRSC07116; DRSC23713	3.57; 5.29
FBgn0015283	Rpn10	DRSC11876	4.34
FBgn0028686	Rpt3	DRSC23412	3.58
FBgn0266666	Sem1	DRSC02282	3.50
FBgn0003392	shi	DRSC20373; DRSC29498	5.20; 5.03
FBgn0019890	Smg5	DRSC03124	3.63
FBgn0264357	SNF4Agamma	DRSC16847; DRSC40676	4.95; 4.12
FBgn0011715	Snr1	DRSC12369	6.12
FBgn0034175	ste24b	DRSC07315	3.24
FBgn0033902	Tango7	DRSC07142; DRSC26908	3.95; 5.70
FBgn0024921	Trn	DRSC25104	4.88
FBgn0011726	tsr	DRSC04718; DRSC40832	6.64; 6.60
FBgn0035124	ttm2	DRSC08268	3.84
FBgn0039530	Tusp	DRSC15838	3.17
FBgn0023143	Uba1	DRSC07567	4.15
FBgn0263697	Uba3	DRSC06377	3.27
FBgn0035853	UbcE2M	DRSC10828	6.22

The genes listed are the ‘hits’ that increased GBP-mediated Ca^2+^ mobilization (i.e., Z-score < − 3), after filtering of housekeeping genes (see “Methods”)

#### Independent validation and additional screening

Follow-up GBP-mediated Ca^2+^ mobilization in S3 cells, using unique dsRNA constructs different from those used in the high-throughput assays, were performed exactly as described previously [5], using 50 nM GBP, and dsRNAs constructed using the following primers (for cDNA fragment and T7-cDNA, respectively):

*Act5c*-f, ATGTGTGACGAAGAAGTTGCTG; TAATACGACTCACTATAGGGTGTGACGAAGAAGTTGCTGC

*Act5c*-r, AAGCCTCCATTCCCAAGAACG; TAATACGACTCACTATAGGGCTGGGTCATCTTCTCACGGT

*Itp*-*r83A*-f, CCTCAAGCGTTTGCATCATGC; TAATACGACTCACTATAGGGGGGCACCTCAATCCAATATG

*Itp*-*r83A*-r, CTGTTTTCCCTTGGGTTTGTCATTTATG; TAATACGACTCACTATAGGGTATGGTGGAGTTCATGGTCG

*Mthl10*-f GCCATAGGCTCTTTCCCAAC; TAATACGACTCACTATAGGGTAGTTCCCGCAGAATTGGTC

Mthl10-r CGTTGACGTATGTCGGAACC; TAATACGACTCACTATAGGGTATGTCGGAACCATGCAGAA

*NorpA*-f, GCGGACTCCTCAAACTATATGC; TAATACGACTCACTATAGGGCTGCCAGATGGTCTCACTCA

*NorpA*-r, GCTCTGCTCCTCAATGCCAAG; TAATACGACTCACTATAGGGGAAGTCCTCAAAGCCGTCA.

*Plc21C*-f, ACGGGAACTGACCTCGATTAG; TAATACGACTCACTATAGGGCACTGCTAAGGGGAATCCAA

*Plc21C*-r, TTGGAGCTTTGTAACGACTAGG; TAATACGACTCACTATAGGGCTGCCAGATGGTCTCACTCA

*Tsr*-f, AGAAATGCGGACCTGGAGAG; TAATACGACTCACTATAGGGCCCAGACCCATCGAAACTAA

*Tsr*-r, CAAATTGGCGATCTCAACAGG; TAATACGACTCACTATAGGGAGGATACGTGTTTCCATCGC

### Results and discussion

The GBP/PLC signaling axis, which acts through of a G-protein coupled receptor (GPCR), stimulates Ca^2+^ mobilization by a biphasic process; first, Ca^2+^ is released from the endoplasmic reticulum, which secondarily stimulates Ca^2+^ entry into the cell [5, 7]. To screen novel genes that regulate the entire Ca^2+^-signaling process, we deployed a genome-wide dsRNA library (the DRSC/TRiP Functional Genomics Resources; https://fgr.hms.harvard.edu/). These dsRNAs were tested using a strain of *Drosophila* S3 cells that encode a fluorescent Ca^2+^ sensor, the *GCaMP3* gene [5].

We screened all 66 library plates at least once, and obtained data for dsRNA knockdown of every gene in the screen (see “Methods”). A scatter-plot of those data that had replicates (see “Methods”) indicate that most amplicons show good reproducibility (Fig. 1b). From the entire data set (Fig. 1c), we found that total Ca^2+^ mobilization ([Ca^2+^]_T_) was inhibited by 103 amplicons (Z score < − 3; Table 1). A separate group of 80 amplicons (Table 2) increased [Ca^2+^]_T_ (Z score > 3). All of these data for each individual dsRNA are available at: http://www.flyrnai.org/screensummary). These numbers of amplicons in the two categories were reduced to 82 and 61, respectively (Tables 1 and 2), after we filtered out housekeeping genes (see “Methods”).

Any high throughput screen is susceptible to false positive and false-negative hits. We were concerned that *plc21C* and *NorpA* may have been false-negatives; neither of these genes were hits in our screen, yet being that they are orthologs of mammalian *PLC*-*β*, one or both of these gene products was expected to mediate GBP-dependent, GPCR-coupled Ca^2+^ mobilization. Thus, we performed additional validation assays using our own, unique dsRNA constructs. Both *plc21C* and *NorpA* were hits in these follow-up assays (Fig. 1d); knock-down of either significantly reduced Ca^2+^ mobilization. The simultaneous knockdown of both genes elicited an approximately additive effect (Fig. 1d). These data suggest partial functional redundancy of the two *PLC*-*β* genes, which can account for their being false negatives in a dsRNA screen.

As mentioned above, we separated our hits into two groups, based on whether [Ca^2+^]_T_ was either decreased (Table 1) or increased (Table 2). We selected representatives from each group for validation. We used our unique dsRNAs at an early stage of this project to validate that knockdown of the *Itp*-*r83A* reduced [Ca^2+^]_T_ (see “Methods”, and Figs. 1a, d); we subsequently deployed *Itp*-*r83A* as a positive control to interrogate dsRNA plate integrity (see “Methods”). A second hit, *Mthl10* (Table 1), was also validated in secondary assays with our own, independent dsRNAs (Fig. 1d).

Among hits that elevate [Ca^2+^]_T_ (Table 2), we selected two—*Tsr* and *Act5C*—for further testing with our independent dsRNAs; in both cases, we confirmed that knockdown of either gene significantly increased [Ca^2+^]_T_. These data indicate that both of these genes normally constrain [Ca^2+^]_T_; it should be interesting to study further the biological significance of such a phenomenon.

Another aspect of our data that is of interest is the determination that GBP-dependent Ca^2+^ signaling is regulated by a family of genes that encode proteins that are components of the proteosome (PSMA2, PSMB6, PSMD6, Rpn11/PSMD14; Tables 1, 2). This multiprotein complex can regulate PLC/GPCR signaling by controlling the cell-surface levels of the receptor [10]; our data highlight the likely participation of the proteosome in regulating the activity of the GBP/Mthl10 signaling axis. Another hit, Gqα (Table 1), is a subunit of a heterotrimeric G-protein that couples GPCRs to the activation of PLC-*β.* These are all data that underscore the value of a systems-level approach to fully understanding all aspects of the Ca^2+^-signaling process.

We propose that human orthologs of our complete list of filtered gene hits (Tables 1 and 2) are not only candidates for acting in molecular pathways that interconnect aging and inflammation, but also potential new modulators of Ca^2+^ signaling in general. Thus, our data may drive several new, future research directions.

## Limitations

A limitation in this study—as is the case for all high throughput screening exercises—is the possibility of false positives and false negatives. The possibility that gene redundancy may lead to false negatives is highlighted by Fig. 1d. In a genome-wide study such as this, it is not feasible to validate every hit, so false positives remain a possibility for future studies that pursue our data.

## Abbreviations

[Ca^2+^]_T_: total calcium mobilization
GBP: growth-blocking peptide
GPCR: G-protein coupled receptor
PLC: phospholipase C
